# 13-Benzyl-4,11-dihy­droxy-1,8-diphen­yl-2,9-di­thia-13-aza­dispiro­[4.1.4.3]tetra­decan-6-one

**DOI:** 10.1107/S2414314621002108

**Published:** 2021-02-26

**Authors:** G. Vinotha, T. V. Sundar, N. Sharmila

**Affiliations:** a Postgraduate and Research Department of Physics, National College (Autonomous), Tiruchirappalli-620001, Tamil Nadu, India; b Department of Physics, Shrimati Indira Gandhi College, Tiruchirappalli-620002, Tamil Nadu, India; Howard University, USA

**Keywords:** crystal structure, Hirshfeld surface, O—H⋯O hydrogen bonds, C⋯H and H⋯H contacts

## Abstract

In the title compound, C_30_H_31_NO_3_S_2_, the piperidine ring adopts a distorted chair conformation. The thio­phene rings have twisted conformations about the C—C bonds. The mean plane of the piperidine ring makes a near orthogonal conformation with the toluene ring. Two of the phenyl rings in the structure are positionally disordered over two sets of sites with occupancies of 0.56 (2)/0.44 (2) and 0.672 (16)/0.328 (16). A region of disordered electron density was corrected for using the SQUEEZE [Spek (2015 ▸). *Acta Cryst.* C**71**, 9–18] routine in *PLATON*. The given chemical formula and other crystal data do not take into account the unknown solvent mol­ecule. In the crystal, O—H⋯O hydrogen bonds are observed along with intra­molecular S⋯H, O⋯H, C⋯H and H⋯H contacts.

## Abstract 2

In the title compound, C_30_H_31_NO_3_S_2_, the piperidine ring adopts a distorted chair conformation. The thio­phene rings have twisted conformations about the C—C bonds. Two of the phenyl rings in the structure are positionally disordered over two sets of sites. The crystal packing features inter­molecular O—H⋯O hydrogen bonds and S⋯H, O⋯H, C⋯H and H⋯H contacts.

## Graphical abstract

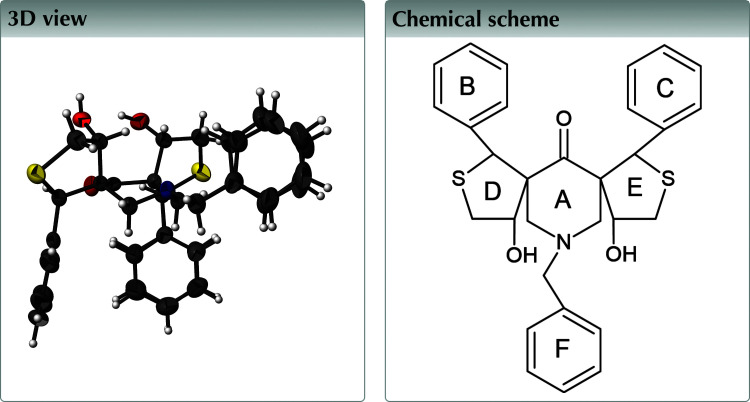

## Structure description

Many substituted piperidine derivatives possess a wide range of bioactivities (Pati & Banerjee, 2012 ▸). They find significant applications in drug development and their properties depend on the nature of the side groups and their orientations (Viswanathan *et al.*, 2015 ▸). As part of our studies in this area, we herein report the crystal structure of the title compound.

**Figure 1:**
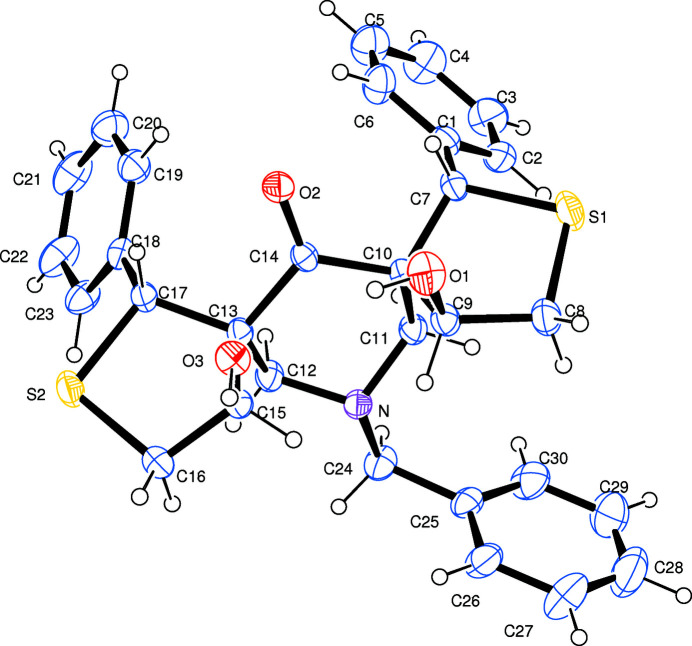
The mol­ecular structure of the title compound with the atom-numbering scheme. Displacement ellipsoids are drawn at the 30% probability level. H atoms are shown as circles of arbitrary radii. The minor conformations of the two disordered phenyl rings are not shown here for clarity.

**Figure 2:**
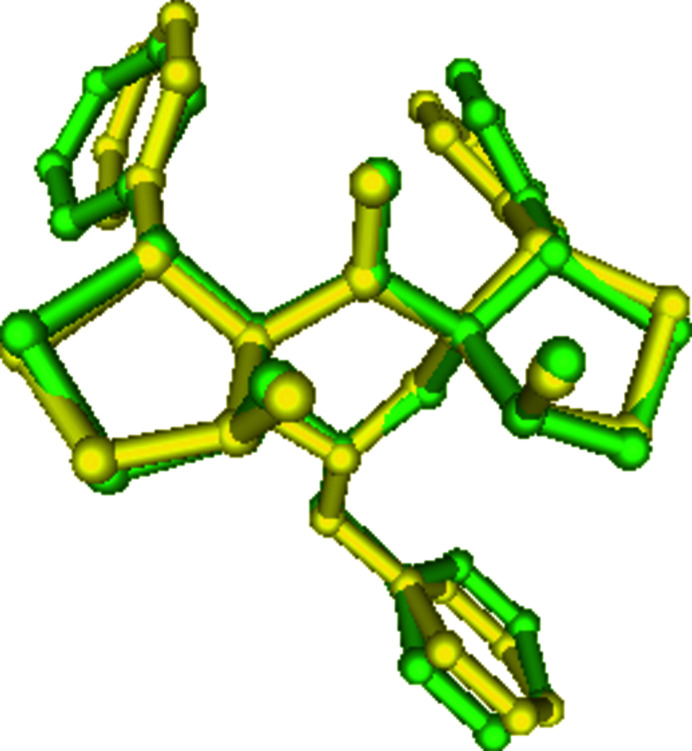
An overlay of similar atoms of the title mol­ecule (green) and the related compound **2** (yellow), excluding H atoms, indicating their nearly identical conformations.

The mol­ecular structure of the title compound with atom numbering is shown in Fig. 1 ▸. The piperidine ring adopts a distorted chair conformation as observed in a similar related structure, **2** {13-benzyl-4,11-dihy­droxy-1,8-bis­(4-methyl­phen­yl)-2,9-di­thia-13-aza­dispiro­[4.1.47.35]tetra­decan-6-one; Viswanathan *et al.*, 2015 ▸}. However, both the thio­phene rings (rings *D* S2/C16/C15/C13/C17 and *E*: S1/C7/C10/C9/C8) have twisted conformations about the C—C bonds (C10—C9 in *D* and C13—C15 in *E*). In **2**, ring *D* adopts an envelope conformation and ring *E* a twisted conformation about the C13—C17 bond, indicating the influence of substitutional effects on the ring conformations. The mean plane of the piperidine ring *A* is nearly orthogonal [88.5 (3)°] to the toluene ring *F*. This angle is reported to be 75.09 (1)° in **2** (Viswanathan *et al.*, 2015 ▸). In addition, the dihedral angles between the mean planes of rings *D* and *B* and between *E* and *C* are 54.07 (14) and 40.5 (4)°, respectively, differing significantly from the values reported for **2**. An overlay analysis of the title compound (with major conformer only) for non-H atoms with the corresponding atoms in **2** has an r.m.s. deviation of 1.12 Å (Fig. 2 ▸). A similar analysis for a compound closely related to **2** (with a methyl rather than a benzyl substituent on the N atom of the central piperidine ring), the r.m.s. deviation is found to be 1.03 Å, indicating the conformational preservation of the five rings (*A* to *E*) in these structures. An intra­molecular O—H⋯O contact (Table 1 ▸) is observed. The phenyl rings attached to rings *D* and *E* are both positionally disordered over two sets of sites with occupancies of 0.56 (2)/0.44 (2) and 0.672 (16)/0.328 (16), respectively.

**Figure 3:**
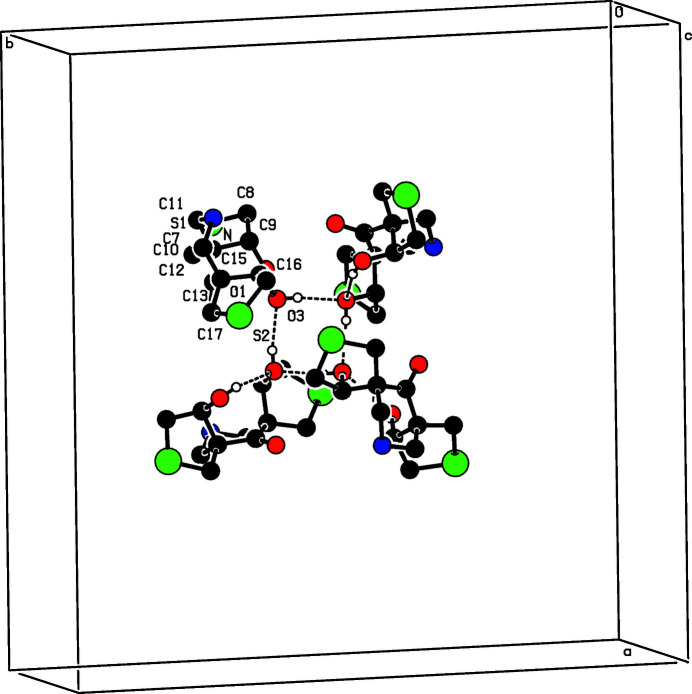
An O—H⋯O inter­action between atoms O3 and O1 of a symmetry-related mol­ecule (at 1 − *x*, −1 + *y* and 1 − *z*) leading to an 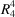 (8) ring motif.

In the crystal, an O—H⋯O inter­actions forms an 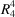 (8) ring motif (Bernstein *et al.*, 1995 ▸). This pattern is stacked along the *c*-axis direction, forming hollow square frames parallel to *c* (Fig. 3 ▸).

**Figure 4:**
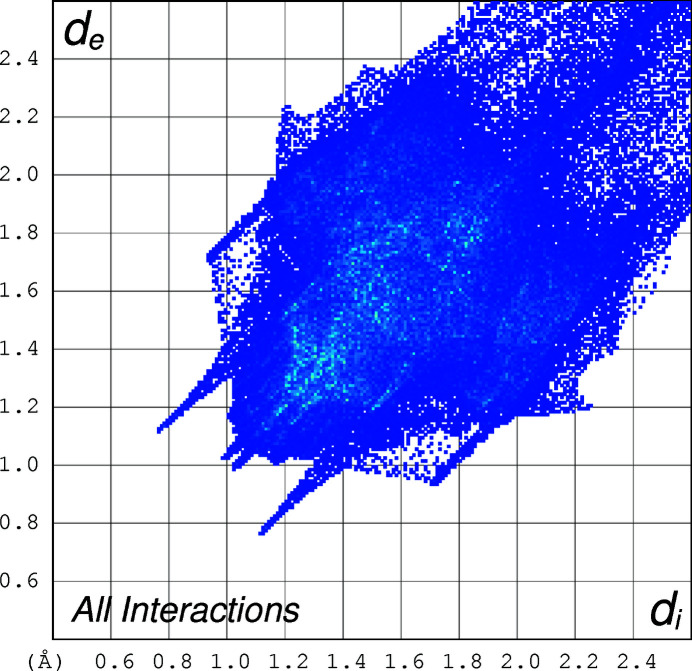
The two-dimensional fingerprint plot for the title compound depicting the overall contribution by the various contacts.

The two-dimensional fingerprint plots (Spackman & Jayatilaka, 2009 ▸) of the mol­ecule, created using *CrystalExplorer17* (Turner *et al.*, 2017 ▸) for the contacts contributing to the Hirshfeld surface, are shown in Figs. 4 ▸–6 ▸ ▸. The analysis reveals that H⋯H contacts (68.2%) and C⋯H contacts (14.3%) are the main contributors to the crystal packing, followed by S⋯H (8.4%) and O⋯H (7.1%) contacts.

## Synthesis and crystallization

A mixture of (3*E*,5*E*)-1-benzyl-3,5-di­benzyl­idenepiperidin-4-one (1 mmol), 1,4-di­thiane-2,5-diol (1 mmol and tri­ethyl­amine (0.25 eq) in di­chloro­methane (6 ml) was heated under reflux for 3 h. After completion of the reaction (TLC), the solvent was removed and the product was purified by flash column using a petroleum ether–ethyl acetate mixture (4:1 *v*/*v*) as eluent (yield 74%, m.p. 476 K). Chloro­form was used as the solvent to harvest crystals for experiment.

## Refinement

Crystal data, data collection and structure refinement details are summarized in Table 2 ▸. A region of disordered electron density (48 e Å^−3^) located near a symmetry element (0.0 0.5 − 0.018) was corrected for using the SQUEEZE (Spek, 2015 ▸) routine in *PLATON*. Two phenyl rings in the modelled structure are found to be positionally disordered over two sets of sites with occupancies of 0.56 (2)/0.44 (2) and 0.672 (16)/0.328 (16). Hence, disorder treatment was applied with the rigid-bond restraints SIMU and DELU for completing the refinement.

## Supplementary Material

Crystal structure: contains datablock(s) I, global. DOI: 10.1107/S2414314621002108/bv4035sup1.cif

Structure factors: contains datablock(s) I. DOI: 10.1107/S2414314621002108/bv4035Isup2.hkl

Supporting information file. DOI: 10.1107/S2414314621002108/bv4035Isup3.cml

CCDC reference: 2064599

Additional supporting information:  crystallographic information; 3D view; checkCIF report

## Figures and Tables

**Figure 5:**
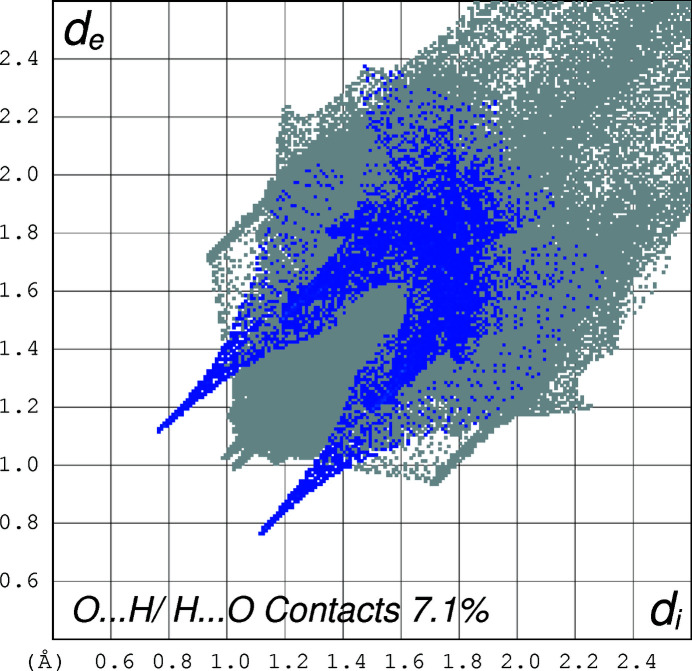
The two-dimensional fingerprint plot for the title compound depicting the contribution percentage of O⋯H contacts.

**Figure 6:**
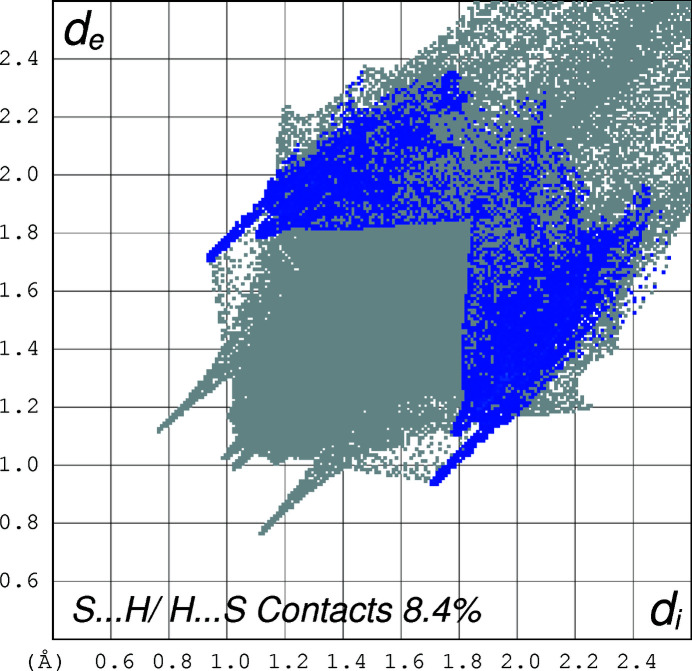
The two-dimensional fingerprint plot for the title compound depicting the contribution percentage of S⋯H contacts.

**Table 1:** Hydrogen-bond geometry (Å, °)

*D*—H⋯*A*	*D*—H	H⋯*A*	*D*⋯*A*	*D*—H⋯*A*
O1—H1⋯O3	0.82	2.31	3.058 (3)	152
O3—H3⋯O3^i^	0.82	2.05	2.851 (3)	167

Symmetry code: (i) 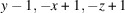 .

**Table 2:** Experimental details

Crystal data	
Chemical formula	C_30_H_31_NO_3_S_2_
*M*_r_	517.68
Crystal system, space group	Tetragonal, *P* 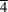 2_1_ *c*
Temperature (K)	293
*a*, *c* (Å)	25.3750 (4), 8.6456 (2)
*V* (Å^3^)	5566.8 (2)
*Z*	8
Radiation type	Mo *K*α
μ (mm^−1^)	0.22
Crystal size (mm)	0.28 × 0.24 × 0.20

Data collection	
Diffractometer	Bruker *SMART* APEXII area-detector
Absorption correction	Multi-scan (*SADABS*; Sheldrick, 1996 ▸)
*T*_min_, *T*_max_	0.685, 0.742
No. of measured, independent and observed [*I* > 2σ(*I*)] reflections	30401, 6899, 5681
*R*_int_	0.033
(sin θ/λ)_max_ (Å^−1^)	0.667

Refinement	
*R*[*F* ^2^ > 2σ(*F* ^2^)], *wR*(*F* ^2^), *S*	0.043, 0.110, 1.03
No. of reflections	6899
No. of parameters	389
No. of restraints	408
H-atom treatment	H-atom parameters constrained
Δρ_max_, Δρ_min_ (e Å^−3^)	0.27, −0.15
Absolute structure	Flack *x* determined using 2151 quotients [(*I* ^+^)−(*I* ^−^)]/[(*I* ^+^)+(*I* ^−^)] (Parsons *et al.*, 2013 ▸)
Absolute structure parameter	−0.04 (2)

Computer programs: *APEX2* and *SAINT* (Bruker, 2008 ▸), *SHELXS97* (Sheldrick, 2008 ▸), *SHELXL* (Sheldrick, 2015 ▸), *ORTEP-3 for Windows* (Farrugia, 2012 ▸), *Qmol* (Gans & Shalloway, 2001 ▸) and *PLATON* (Spek, 2020 ▸)’.

## full crystallographic data

### Crystal data

**Table:**

C_30_H_31_NO_3_S_2_	Melting point: 476.15 K
*M**_r_* = 517.68	Mo *K*α radiation, λ = 0.71073 Å
Tetragonal, *P*42_1_*c*	Cell parameters from 6903 reflections
*a* = 25.3750 (4) Å	θ = 1.1–28.3°
*c* = 8.6456 (2) Å	µ = 0.22 mm^−^^1^
*V* = 5566.8 (2) Å^3^	*T* = 293 K
*Z* = 8	Block, colourless
*F*(000) = 2192	0.28 × 0.24 × 0.20 mm
*D*_x_ = 1.235 Mg m^−^^3^	

### Data collection

**Table:**

Bruker SMART APEXII area-detector diffractometer	5681 reflections with *I* > 2σ(*I*)
ω and φ scans	*R*_int_ = 0.033
Absorption correction: multi-scan (SADABS; Sheldrick, 1996)	θ_max_ = 28.3°, θ_min_ = 1.8°
*T*_min_ = 0.685, *T*_max_ = 0.742	*h* = −33→23
30401 measured reflections	*k* = −33→27
6899 independent reflections	*l* = −11→11

### Refinement

**Table:**

Refinement on *F*^2^	Secondary atom site location: difference Fourier map
Least-squares matrix: full	Hydrogen site location: inferred from neighbouring sites
*R*[*F*^2^ > 2σ(*F*^2^)] = 0.043	H-atom parameters constrained
*wR*(*F*^2^) = 0.110	*w* = 1/[σ^2^(*F*_o_^2^) + (0.0659*P*)^2^ + 0.1922*P*] where *P* = (*F*_o_^2^ + 2*F*_c_^2^)/3
*S* = 1.02	(Δ/σ)_max_ = 0.001
6899 reflections	Δρ_max_ = 0.27 e Å^−^^3^
389 parameters	Δρ_min_ = −0.15 e Å^−^^3^
408 restraints	Absolute structure: Flack *x* determined using 2151 quotients [(*I*^+^)-(*I*^-^)]/[(*I*^+^)+(*I*^-^)] (Parsons *et al.*, 2013)
Primary atom site location: structure-invariant direct methods	Absolute structure parameter: −0.04 (2)

### Special details

**Table:**

Geometry. All esds (except the esd in the dihedral angle between two l.s. planes) are estimated using the full covariance matrix. The cell esds are taken into account individually in the estimation of esds in distances, angles and torsion angles; correlations between esds in cell parameters are only used when they are defined by crystal symmetry. An approximate (isotropic) treatment of cell esds is used for estimating esds involving l.s. planes.
Refinement. The hydroxy H atoms were refined as riding: O—H = 0.82 Å with *U*_iso_(H) = 1.5*U*_eq_(O)·. The C-bound H atoms were included in calculated positions and treated as riding, with C—H = 0.95–0.98 Å, and with 1.2*U*_eq_(C) for H atoms.

### Fractional atomic coordinates and isotropic or equivalent isotropic displacement parameters (Å^2^)

**Table:**

	*x*	*y*	*z*	*U*_iso_*/*U*_eq_	Occ. (<1)
S1	0.19875 (3)	0.80219 (4)	0.82727 (10)	0.0581 (2)	
S2	0.07519 (3)	0.97740 (3)	0.14696 (9)	0.04708 (19)	
N	0.13618 (9)	0.81533 (8)	0.3251 (2)	0.0365 (5)	
O1	0.10182 (9)	0.88166 (9)	0.7697 (2)	0.0528 (5)	
H1	0.083927	0.903237	0.722551	0.079*	
O2	0.17096 (8)	0.94891 (7)	0.5606 (2)	0.0440 (5)	
O3	0.05248 (7)	0.94022 (8)	0.4969 (2)	0.0414 (4)	
H3	0.020531	0.939159	0.510786	0.062*	
C1	0.2681 (4)	0.8548 (5)	0.6236 (10)	0.043 (3)	0.44 (2)
C2	0.3011 (6)	0.8117 (5)	0.6461 (15)	0.051 (2)	0.44 (2)
H2A	0.289787	0.783422	0.705944	0.061*	0.44 (2)
C3	0.3509 (6)	0.8109 (5)	0.5791 (16)	0.065 (2)	0.44 (2)
H3A	0.372892	0.782049	0.594152	0.078*	0.44 (2)
C4	0.3677 (4)	0.8532 (5)	0.4897 (13)	0.074 (2)	0.44 (2)
H4A	0.401038	0.852616	0.444862	0.089*	0.44 (2)
C5	0.3348 (5)	0.8963 (5)	0.4672 (18)	0.067 (2)	0.44 (2)
H5A	0.346080	0.924556	0.407363	0.081*	0.44 (2)
C6	0.2850 (4)	0.8971 (5)	0.5342 (17)	0.057 (2)	0.44 (2)
H6A	0.262974	0.925931	0.519155	0.068*	0.44 (2)
C1A	0.2679 (3)	0.8540 (4)	0.6274 (8)	0.0401 (19)	0.56 (2)
C2A	0.2951 (4)	0.8070 (3)	0.6069 (13)	0.0478 (18)	0.56 (2)
H2AA	0.279047	0.775147	0.631921	0.057*	0.56 (2)
C3A	0.3462 (4)	0.8075 (3)	0.5489 (12)	0.066 (2)	0.56 (2)
H3AA	0.364329	0.776020	0.535225	0.079*	0.56 (2)
C4A	0.3701 (3)	0.8551 (4)	0.5115 (11)	0.079 (2)	0.56 (2)
H4AA	0.404302	0.855444	0.472747	0.094*	0.56 (2)
C5A	0.3430 (4)	0.9022 (4)	0.5320 (17)	0.072 (2)	0.56 (2)
H5AA	0.358993	0.933996	0.506964	0.087*	0.56 (2)
C6A	0.2919 (3)	0.9016 (3)	0.5899 (14)	0.0524 (18)	0.56 (2)
H6AA	0.273711	0.933124	0.603660	0.063*	0.56 (2)
C7	0.21172 (10)	0.85613 (11)	0.6917 (3)	0.0370 (6)	
H7	0.208163	0.889184	0.749387	0.044*	
C8	0.13063 (12)	0.79323 (13)	0.7676 (4)	0.0495 (7)	
H8A	0.126873	0.760935	0.708600	0.059*	
H8B	0.107757	0.791298	0.857322	0.059*	
C9	0.11644 (10)	0.84041 (10)	0.6689 (3)	0.0381 (6)	
H9	0.086970	0.831599	0.600358	0.046*	
C10	0.16622 (10)	0.85436 (10)	0.5712 (3)	0.0319 (5)	
C11	0.17586 (11)	0.81241 (10)	0.4471 (3)	0.0380 (6)	
H11A	0.210565	0.817450	0.402454	0.046*	
H11B	0.174908	0.777734	0.494223	0.046*	
C12	0.14383 (11)	0.86442 (10)	0.2399 (3)	0.0381 (6)	
H12A	0.123068	0.863910	0.145687	0.046*	
H12B	0.180634	0.868270	0.211807	0.046*	
C13	0.12668 (9)	0.91073 (9)	0.3422 (3)	0.0314 (5)	
C14	0.15673 (9)	0.90880 (10)	0.4977 (3)	0.0315 (5)	
C15	0.06627 (10)	0.90622 (10)	0.3714 (3)	0.0352 (5)	
H15	0.057208	0.869749	0.397543	0.042*	
C16	0.03912 (11)	0.92192 (12)	0.2229 (4)	0.0457 (7)	
H16A	0.002780	0.931643	0.242898	0.055*	
H16B	0.039538	0.892939	0.149758	0.055*	
C17	0.13408 (10)	0.96560 (10)	0.2640 (3)	0.0350 (5)	
H17	0.133754	0.991842	0.347127	0.042*	
C18	0.18491 (10)	0.97408 (9)	0.1735 (3)	0.0374 (5)	
C19	0.22921 (12)	0.99325 (12)	0.2497 (4)	0.0505 (7)	
H19	0.227904	0.998813	0.355936	0.061*	
C20	0.27490 (14)	1.00411 (13)	0.1705 (5)	0.0645 (9)	
H20	0.304160	1.016710	0.223658	0.077*	
C21	0.27770 (16)	0.99650 (13)	0.0129 (5)	0.0681 (10)	
H21	0.308518	1.004157	−0.040764	0.082*	
C22	0.23375 (16)	0.97718 (14)	−0.0645 (4)	0.0664 (10)	
H22	0.235172	0.972066	−0.170956	0.080*	
C23	0.18816 (13)	0.96554 (12)	0.0146 (3)	0.0499 (7)	
H23	0.159320	0.951888	−0.038361	0.060*	
C24	0.13841 (13)	0.76884 (11)	0.2234 (3)	0.0473 (7)	
H24A	0.174111	0.764142	0.185664	0.057*	
H24B	0.115547	0.774237	0.134857	0.057*	
C25	0.1210 (3)	0.71931 (18)	0.3115 (8)	0.0449 (15)	0.672 (16)
C26	0.0695 (2)	0.7125 (3)	0.3626 (11)	0.0587 (17)	0.672 (16)
H26A	0.044391	0.738401	0.343379	0.070*	0.672 (16)
C27	0.0556 (2)	0.6671 (3)	0.4425 (10)	0.086 (2)	0.672 (16)
H27A	0.021107	0.662541	0.476661	0.103*	0.672 (16)
C28	0.0931 (4)	0.6284 (2)	0.4712 (9)	0.097 (2)	0.672 (16)
H28A	0.083765	0.597997	0.524630	0.116*	0.672 (16)
C29	0.1446 (3)	0.6352 (2)	0.4201 (10)	0.089 (2)	0.672 (16)
H29A	0.169708	0.609312	0.439317	0.106*	0.672 (16)
C30	0.1585 (3)	0.6806 (2)	0.3402 (9)	0.0669 (17)	0.672 (16)
H30A	0.192993	0.685172	0.306033	0.080*	0.672 (16)
C25A	0.1208 (6)	0.7211 (4)	0.2988 (18)	0.048 (3)	0.328 (16)
C26A	0.0731 (6)	0.7254 (5)	0.377 (2)	0.054 (3)	0.328 (16)
H26B	0.056243	0.757823	0.383767	0.065*	0.328 (16)
C27A	0.0508 (5)	0.6812 (6)	0.446 (2)	0.080 (3)	0.328 (16)
H27B	0.018955	0.684108	0.498750	0.096*	0.328 (16)
C28A	0.0761 (6)	0.6327 (5)	0.4366 (19)	0.089 (3)	0.328 (16)
H28B	0.061129	0.603172	0.482718	0.106*	0.328 (16)
C29A	0.1237 (7)	0.6284 (4)	0.358 (2)	0.086 (3)	0.328 (16)
H29B	0.140592	0.595950	0.351704	0.103*	0.328 (16)
C30A	0.1460 (5)	0.6726 (5)	0.2892 (19)	0.069 (3)	0.328 (16)
H30B	0.177882	0.669665	0.236720	0.083*	0.328 (16)

### Atomic displacement parameters (Å^2^)

**Table:**

	*U*^11^	*U*^22^	*U*^33^	*U*^12^	*U*^13^	*U*^23^
S1	0.0511 (4)	0.0731 (5)	0.0502 (4)	−0.0028 (4)	−0.0115 (4)	0.0292 (4)
S2	0.0453 (4)	0.0521 (4)	0.0438 (4)	0.0018 (3)	−0.0063 (3)	0.0186 (3)
N	0.0486 (12)	0.0305 (10)	0.0305 (11)	−0.0012 (9)	−0.0034 (10)	−0.0029 (9)
O1	0.0582 (13)	0.0577 (13)	0.0424 (11)	0.0094 (10)	0.0105 (10)	−0.0008 (10)
O2	0.0581 (12)	0.0362 (10)	0.0377 (10)	−0.0013 (8)	−0.0104 (9)	−0.0039 (8)
O3	0.0374 (10)	0.0483 (11)	0.0386 (10)	0.0014 (8)	0.0034 (8)	0.0008 (9)
C1	0.039 (5)	0.044 (5)	0.046 (5)	0.005 (4)	−0.009 (4)	0.001 (4)
C2	0.046 (4)	0.045 (4)	0.060 (5)	−0.002 (3)	−0.010 (4)	0.003 (4)
C3	0.047 (4)	0.065 (4)	0.084 (5)	0.011 (4)	0.000 (4)	−0.004 (4)
C4	0.051 (4)	0.080 (5)	0.091 (5)	0.006 (4)	0.016 (4)	0.000 (4)
C5	0.054 (4)	0.072 (4)	0.076 (6)	−0.002 (3)	0.009 (5)	0.009 (5)
C6	0.049 (4)	0.053 (4)	0.068 (6)	0.005 (3)	0.006 (4)	0.013 (4)
C1A	0.037 (4)	0.043 (4)	0.040 (4)	0.002 (3)	−0.006 (3)	0.002 (3)
C2A	0.043 (3)	0.049 (3)	0.051 (4)	0.002 (2)	−0.013 (3)	−0.008 (3)
C3A	0.050 (3)	0.065 (4)	0.081 (4)	0.016 (3)	−0.005 (3)	−0.012 (3)
C4A	0.049 (4)	0.084 (4)	0.102 (5)	0.007 (3)	0.019 (4)	0.004 (4)
C5A	0.052 (4)	0.072 (4)	0.092 (6)	−0.001 (3)	0.014 (4)	0.016 (4)
C6A	0.047 (3)	0.048 (3)	0.062 (5)	0.009 (3)	0.003 (3)	0.010 (3)
C7	0.0398 (13)	0.0405 (13)	0.0308 (13)	0.0000 (11)	−0.0042 (10)	0.0058 (11)
C8	0.0467 (16)	0.0555 (17)	0.0464 (16)	−0.0029 (13)	−0.0034 (13)	0.0175 (14)
C9	0.0404 (13)	0.0428 (13)	0.0310 (13)	−0.0013 (11)	0.0000 (11)	0.0049 (12)
C10	0.0346 (12)	0.0328 (12)	0.0282 (12)	−0.0003 (9)	−0.0023 (10)	0.0027 (10)
C11	0.0426 (14)	0.0332 (13)	0.0382 (13)	0.0037 (10)	−0.0015 (11)	0.0006 (11)
C12	0.0484 (15)	0.0357 (13)	0.0302 (12)	0.0013 (11)	0.0003 (12)	−0.0004 (11)
C13	0.0350 (12)	0.0318 (11)	0.0272 (11)	−0.0003 (9)	−0.0006 (10)	0.0029 (10)
C14	0.0319 (12)	0.0372 (13)	0.0254 (11)	0.0021 (10)	0.0012 (10)	0.0013 (10)
C15	0.0355 (13)	0.0378 (13)	0.0324 (13)	−0.0028 (10)	−0.0025 (11)	0.0040 (11)
C16	0.0442 (15)	0.0496 (16)	0.0432 (16)	−0.0022 (12)	−0.0107 (12)	0.0070 (13)
C17	0.0396 (13)	0.0340 (12)	0.0313 (12)	0.0015 (10)	−0.0037 (11)	0.0042 (10)
C18	0.0454 (14)	0.0285 (12)	0.0383 (13)	0.0009 (10)	0.0030 (11)	0.0043 (11)
C19	0.0462 (16)	0.0539 (17)	0.0514 (17)	−0.0058 (13)	0.0004 (14)	0.0103 (15)
C20	0.0486 (18)	0.061 (2)	0.084 (3)	−0.0088 (15)	−0.0008 (19)	0.0099 (19)
C21	0.066 (2)	0.0541 (19)	0.085 (3)	−0.0054 (16)	0.038 (2)	0.0046 (19)
C22	0.089 (3)	0.0534 (19)	0.057 (2)	−0.0153 (18)	0.031 (2)	−0.0065 (17)
C23	0.0624 (18)	0.0438 (15)	0.0436 (16)	−0.0117 (13)	0.0110 (14)	−0.0027 (13)
C24	0.0670 (19)	0.0379 (14)	0.0370 (15)	−0.0026 (13)	0.0065 (14)	−0.0078 (12)
C25	0.053 (3)	0.034 (3)	0.047 (3)	−0.006 (2)	0.001 (3)	−0.007 (2)
C26	0.054 (3)	0.042 (3)	0.081 (4)	−0.009 (3)	0.001 (3)	0.001 (3)
C27	0.089 (4)	0.060 (5)	0.110 (4)	−0.021 (3)	0.024 (4)	0.008 (4)
C28	0.127 (5)	0.055 (3)	0.109 (5)	−0.014 (4)	0.004 (4)	0.023 (3)
C29	0.109 (5)	0.045 (3)	0.112 (5)	0.014 (3)	−0.017 (4)	0.005 (3)
C30	0.071 (3)	0.041 (3)	0.089 (4)	0.004 (2)	−0.003 (3)	−0.009 (3)
C25A	0.055 (5)	0.033 (5)	0.056 (5)	0.003 (5)	−0.005 (5)	−0.009 (5)
C26A	0.061 (5)	0.033 (5)	0.070 (5)	−0.018 (4)	0.004 (4)	−0.008 (5)
C27A	0.089 (5)	0.046 (6)	0.106 (6)	−0.014 (4)	0.016 (5)	0.005 (5)
C28A	0.105 (6)	0.050 (5)	0.111 (6)	−0.012 (5)	0.007 (5)	0.024 (5)
C29A	0.102 (6)	0.038 (4)	0.117 (7)	0.006 (5)	−0.007 (6)	0.008 (5)
C30A	0.073 (5)	0.036 (4)	0.098 (6)	0.002 (4)	0.002 (5)	−0.011 (5)

### Geometric parameters (Å, º)

**Table:**

S1—C8	1.818 (3)	C12—H12A	0.9700
S1—C7	1.832 (3)	C12—H12B	0.9700
S2—C16	1.803 (3)	C13—C14	1.546 (3)
S2—C17	1.829 (3)	C13—C15	1.558 (3)
N—C11	1.460 (3)	C13—C17	1.559 (3)
N—C12	1.460 (3)	C15—C16	1.511 (4)
N—C24	1.473 (3)	C15—H15	0.9800
O1—C9	1.412 (3)	C16—H16A	0.9700
O1—H1	0.8200	C16—H16B	0.9700
O2—C14	1.209 (3)	C17—C18	1.524 (4)
O3—C15	1.430 (3)	C17—H17	0.9800
O3—H3	0.8200	C18—C19	1.390 (4)
C1—C2	1.3900	C18—C23	1.394 (4)
C1—C6	1.3900	C19—C20	1.374 (5)
C1—C7	1.548 (10)	C19—H19	0.9300
C2—C3	1.3900	C20—C21	1.377 (6)
C2—H2A	0.9300	C20—H20	0.9300
C3—C4	1.3900	C21—C22	1.390 (6)
C3—H3A	0.9300	C21—H21	0.9300
C4—C5	1.3900	C22—C23	1.376 (5)
C4—H4A	0.9300	C22—H22	0.9300
C5—C6	1.3900	C23—H23	0.9300
C5—H5A	0.9300	C24—C25A	1.448 (10)
C6—H6A	0.9300	C24—C25	1.535 (6)
C1A—C2A	1.3900	C24—H24A	0.9700
C1A—C6A	1.3900	C24—H24B	0.9700
C1A—C7	1.532 (8)	C25—C26	1.3900
C2A—C3A	1.3900	C25—C30	1.3900
C2A—H2AA	0.9300	C26—C27	1.3900
C3A—C4A	1.3900	C26—H26A	0.9300
C3A—H3AA	0.9300	C27—C28	1.3900
C4A—C5A	1.3900	C27—H27A	0.9300
C4A—H4AA	0.9300	C28—C29	1.3900
C5A—C6A	1.3900	C28—H28A	0.9300
C5A—H5AA	0.9300	C29—C30	1.3900
C6A—H6AA	0.9300	C29—H29A	0.9300
C7—C10	1.556 (3)	C30—H30A	0.9300
C7—H7	0.9800	C25A—C26A	1.3900
C8—C9	1.514 (4)	C25A—C30A	1.3900
C8—H8A	0.9700	C26A—C27A	1.3900
C8—H8B	0.9700	C26A—H26B	0.9300
C9—C10	1.560 (4)	C27A—C28A	1.3900
C9—H9	0.9800	C27A—H27B	0.9300
C10—C11	1.531 (3)	C28A—C29A	1.3900
C10—C14	1.540 (3)	C28A—H28B	0.9300
C11—H11A	0.9700	C29A—C30A	1.3900
C11—H11B	0.9700	C29A—H29B	0.9300
C12—C13	1.534 (4)	C30A—H30B	0.9300
			
C8—S1—C7	94.74 (12)	C14—C13—C17	110.24 (19)
C16—S2—C17	94.91 (12)	C15—C13—C17	104.74 (19)
C11—N—C12	108.4 (2)	O2—C14—C10	121.5 (2)
C11—N—C24	111.4 (2)	O2—C14—C13	120.8 (2)
C12—N—C24	112.1 (2)	C10—C14—C13	117.7 (2)
C9—O1—H1	109.5	O3—C15—C16	112.0 (2)
C15—O3—H3	109.5	O3—C15—C13	108.7 (2)
C2—C1—C6	120.0	C16—C15—C13	106.9 (2)
C2—C1—C7	121.3 (8)	O3—C15—H15	109.7
C6—C1—C7	118.6 (8)	C16—C15—H15	109.7
C3—C2—C1	120.0	C13—C15—H15	109.7
C3—C2—H2A	120.0	C15—C16—S2	106.49 (19)
C1—C2—H2A	120.0	C15—C16—H16A	110.4
C4—C3—C2	120.0	S2—C16—H16A	110.4
C4—C3—H3A	120.0	C15—C16—H16B	110.4
C2—C3—H3A	120.0	S2—C16—H16B	110.4
C3—C4—C5	120.0	H16A—C16—H16B	108.6
C3—C4—H4A	120.0	C18—C17—C13	116.8 (2)
C5—C4—H4A	120.0	C18—C17—S2	112.63 (18)
C6—C5—C4	120.0	C13—C17—S2	106.71 (16)
C6—C5—H5A	120.0	C18—C17—H17	106.7
C4—C5—H5A	120.0	C13—C17—H17	106.7
C5—C6—C1	120.0	S2—C17—H17	106.7
C5—C6—H6A	120.0	C19—C18—C23	118.3 (3)
C1—C6—H6A	120.0	C19—C18—C17	119.4 (2)
C2A—C1A—C6A	120.0	C23—C18—C17	122.3 (3)
C2A—C1A—C7	122.5 (6)	C20—C19—C18	121.1 (3)
C6A—C1A—C7	117.5 (6)	C20—C19—H19	119.5
C1A—C2A—C3A	120.0	C18—C19—H19	119.5
C1A—C2A—H2AA	120.0	C19—C20—C21	120.5 (3)
C3A—C2A—H2AA	120.0	C19—C20—H20	119.7
C4A—C3A—C2A	120.0	C21—C20—H20	119.7
C4A—C3A—H3AA	120.0	C20—C21—C22	119.0 (3)
C2A—C3A—H3AA	120.0	C20—C21—H21	120.5
C3A—C4A—C5A	120.0	C22—C21—H21	120.5
C3A—C4A—H4AA	120.0	C23—C22—C21	120.7 (3)
C5A—C4A—H4AA	120.0	C23—C22—H22	119.6
C4A—C5A—C6A	120.0	C21—C22—H22	119.6
C4A—C5A—H5AA	120.0	C22—C23—C18	120.4 (3)
C6A—C5A—H5AA	120.0	C22—C23—H23	119.8
C5A—C6A—C1A	120.0	C18—C23—H23	119.8
C5A—C6A—H6AA	120.0	C25A—C24—N	112.9 (6)
C1A—C6A—H6AA	120.0	N—C24—C25	110.4 (3)
C1A—C7—C10	116.6 (3)	N—C24—H24A	109.6
C1—C7—C10	115.5 (4)	C25—C24—H24A	109.6
C1A—C7—S1	111.9 (4)	N—C24—H24B	109.6
C1—C7—S1	113.1 (5)	C25—C24—H24B	109.6
C10—C7—S1	105.87 (16)	H24A—C24—H24B	108.1
C1—C7—H7	107.3	C26—C25—C30	120.0
C10—C7—H7	107.3	C26—C25—C24	122.0 (4)
S1—C7—H7	107.3	C30—C25—C24	118.0 (4)
C9—C8—S1	106.7 (2)	C27—C26—C25	120.0
C9—C8—H8A	110.4	C27—C26—H26A	120.0
S1—C8—H8A	110.4	C25—C26—H26A	120.0
C9—C8—H8B	110.4	C26—C27—C28	120.0
S1—C8—H8B	110.4	C26—C27—H27A	120.0
H8A—C8—H8B	108.6	C28—C27—H27A	120.0
O1—C9—C8	107.5 (2)	C27—C28—C29	120.0
O1—C9—C10	112.3 (2)	C27—C28—H28A	120.0
C8—C9—C10	107.0 (2)	C29—C28—H28A	120.0
O1—C9—H9	110.0	C30—C29—C28	120.0
C8—C9—H9	110.0	C30—C29—H29A	120.0
C10—C9—H9	110.0	C28—C29—H29A	120.0
C11—C10—C14	111.0 (2)	C29—C30—C25	120.0
C11—C10—C7	111.8 (2)	C29—C30—H30A	120.0
C14—C10—C7	111.51 (19)	C25—C30—H30A	120.0
C11—C10—C9	110.5 (2)	C26A—C25A—C30A	120.0
C14—C10—C9	107.49 (19)	C26A—C25A—C24	115.0 (10)
C7—C10—C9	104.2 (2)	C30A—C25A—C24	124.9 (10)
N—C11—C10	111.2 (2)	C25A—C26A—C27A	120.0
N—C11—H11A	109.4	C25A—C26A—H26B	120.0
C10—C11—H11A	109.4	C27A—C26A—H26B	120.0
N—C11—H11B	109.4	C26A—C27A—C28A	120.0
C10—C11—H11B	109.4	C26A—C27A—H27B	120.0
H11A—C11—H11B	108.0	C28A—C27A—H27B	120.0
N—C12—C13	109.0 (2)	C29A—C28A—C27A	120.0
N—C12—H12A	109.9	C29A—C28A—H28B	120.0
C13—C12—H12A	109.9	C27A—C28A—H28B	120.0
N—C12—H12B	109.9	C28A—C29A—C30A	120.0
C13—C12—H12B	109.9	C28A—C29A—H29B	120.0
H12A—C12—H12B	108.3	C30A—C29A—H29B	120.0
C12—C13—C14	109.70 (19)	C29A—C30A—C25A	120.0
C12—C13—C15	108.5 (2)	C29A—C30A—H30B	120.0
C14—C13—C15	109.98 (19)	C25A—C30A—H30B	120.0
C12—C13—C17	113.6 (2)		
			
C6—C1—C2—C3	0.0	C9—C10—C14—C13	−85.9 (2)
C7—C1—C2—C3	177.8 (7)	C12—C13—C14—O2	143.1 (2)
C1—C2—C3—C4	0.0	C15—C13—C14—O2	−97.7 (3)
C2—C3—C4—C5	0.0	C17—C13—C14—O2	17.3 (3)
C3—C4—C5—C6	0.0	C12—C13—C14—C10	−38.7 (3)
C4—C5—C6—C1	0.0	C15—C13—C14—C10	80.5 (2)
C2—C1—C6—C5	0.0	C17—C13—C14—C10	−164.5 (2)
C7—C1—C6—C5	−177.8 (7)	C12—C13—C15—O3	165.0 (2)
C6A—C1A—C2A—C3A	0.0	C14—C13—C15—O3	45.1 (3)
C7—C1A—C2A—C3A	−179.6 (6)	C17—C13—C15—O3	−73.4 (2)
C1A—C2A—C3A—C4A	0.0	C12—C13—C15—C16	−74.0 (3)
C2A—C3A—C4A—C5A	0.0	C14—C13—C15—C16	166.1 (2)
C3A—C4A—C5A—C6A	0.0	C17—C13—C15—C16	47.6 (3)
C4A—C5A—C6A—C1A	0.0	O3—C15—C16—S2	78.5 (2)
C2A—C1A—C6A—C5A	0.0	C13—C15—C16—S2	−40.4 (3)
C7—C1A—C6A—C5A	179.6 (5)	C17—S2—C16—C15	17.7 (2)
C2A—C1A—C7—C10	−91.3 (6)	C12—C13—C17—C18	−42.0 (3)
C6A—C1A—C7—C10	89.1 (6)	C14—C13—C17—C18	81.6 (3)
C2A—C1A—C7—S1	30.8 (6)	C15—C13—C17—C18	−160.2 (2)
C6A—C1A—C7—S1	−148.8 (6)	C12—C13—C17—S2	85.0 (2)
C2—C1—C7—C10	−112.3 (8)	C14—C13—C17—S2	−151.45 (16)
C6—C1—C7—C10	65.5 (9)	C15—C13—C17—S2	−33.2 (2)
C2—C1—C7—S1	10.0 (9)	C16—S2—C17—C18	139.0 (2)
C6—C1—C7—S1	−172.2 (7)	C16—S2—C17—C13	9.6 (2)
C8—S1—C7—C1A	−143.9 (3)	C13—C17—C18—C19	−88.8 (3)
C8—S1—C7—C1	−143.4 (4)	S2—C17—C18—C19	147.2 (2)
C8—S1—C7—C10	−15.9 (2)	C13—C17—C18—C23	94.2 (3)
C7—S1—C8—C9	−12.3 (2)	S2—C17—C18—C23	−29.8 (3)
S1—C8—C9—O1	−83.4 (2)	C23—C18—C19—C20	0.7 (4)
S1—C8—C9—C10	37.3 (3)	C17—C18—C19—C20	−176.4 (3)
C1A—C7—C10—C11	44.5 (5)	C18—C19—C20—C21	0.4 (5)
C1—C7—C10—C11	45.3 (6)	C19—C20—C21—C22	−0.6 (5)
S1—C7—C10—C11	−80.7 (2)	C20—C21—C22—C23	−0.3 (6)
C1A—C7—C10—C14	−80.5 (5)	C21—C22—C23—C18	1.5 (5)
C1—C7—C10—C14	−79.6 (6)	C19—C18—C23—C22	−1.6 (5)
S1—C7—C10—C14	154.29 (17)	C17—C18—C23—C22	175.4 (3)
C1A—C7—C10—C9	163.8 (4)	C11—N—C24—C25A	69.8 (7)
C1—C7—C10—C9	164.7 (6)	C12—N—C24—C25A	−168.5 (7)
S1—C7—C10—C9	38.6 (2)	C11—N—C24—C25	67.9 (4)
O1—C9—C10—C11	−171.5 (2)	C12—N—C24—C25	−170.4 (3)
C8—C9—C10—C11	70.8 (3)	N—C24—C25—C26	66.9 (5)
O1—C9—C10—C14	−50.2 (3)	N—C24—C25—C30	−112.9 (5)
C8—C9—C10—C14	−167.9 (2)	C30—C25—C26—C27	0.0
O1—C9—C10—C7	68.2 (3)	C24—C25—C26—C27	−179.8 (5)
C8—C9—C10—C7	−49.5 (3)	C25—C26—C27—C28	0.0
C12—N—C11—C10	68.0 (3)	C26—C27—C28—C29	0.0
C24—N—C11—C10	−168.2 (2)	C27—C28—C29—C30	0.0
C14—C10—C11—N	−48.1 (3)	C28—C29—C30—C25	0.0
C7—C10—C11—N	−173.3 (2)	C26—C25—C30—C29	0.0
C9—C10—C11—N	71.1 (3)	C24—C25—C30—C29	179.8 (5)
C11—N—C12—C13	−71.5 (3)	N—C24—C25A—C26A	48.5 (9)
C24—N—C12—C13	165.1 (2)	N—C24—C25A—C30A	−135.6 (10)
N—C12—C13—C14	55.1 (3)	C30A—C25A—C26A—C27A	0.0
N—C12—C13—C15	−65.1 (3)	C24—C25A—C26A—C27A	176.1 (11)
N—C12—C13—C17	178.9 (2)	C25A—C26A—C27A—C28A	0.0
C11—C10—C14—O2	−146.7 (2)	C26A—C27A—C28A—C29A	0.0
C7—C10—C14—O2	−21.3 (3)	C27A—C28A—C29A—C30A	0.0
C9—C10—C14—O2	92.3 (3)	C28A—C29A—C30A—C25A	0.0
C11—C10—C14—C13	35.2 (3)	C26A—C25A—C30A—C29A	0.0
C7—C10—C14—C13	160.5 (2)	C24—C25A—C30A—C29A	−175.7 (12)

### Hydrogen-bond geometry (Å, º)

**Table:**

*D*—H···*A*	*D*—H	H···*A*	*D*···*A*	*D*—H···*A*
O1—H1···O3	0.82	2.31	3.058 (3)	152
O3—H3···O3^i^	0.82	2.05	2.851 (3)	167

Symmetry code: (i) *y*−1, −*x*+1, −*z*+1.
